# Effective Management of Life-Threatening Generalized Pustular Psoriasis Flare With Spesolimab

**DOI:** 10.7759/cureus.64474

**Published:** 2024-07-13

**Authors:** Efterpi Zafiriou, Emmanouil Karampinis, George Giannoulis, Agoritsa Gravani, Stella Gampeta, Kalliopi Zachou

**Affiliations:** 1 Department of Dermatology, Faculty of Medicine, School of Health Sciences, University General Hospital of Larissa, University of Thessaly, Larissa, GRC; 2 Department of Internal Medicine, Faculty of Medicine, School of Health Sciences, University General Hospital of Larissa, University of Thessaly, Larissa, GRC

**Keywords:** psoriasis pathophysiology, treatment choices, il-36, spesolimab, generalized pustular psoriasis (gpp)

## Abstract

Generalized pustular psoriasis (GPP) presents as a severe variant of psoriasis featuring painful, sterile pustules on red skin and can lead to life-threatening complications if left untreated. The disease course is typically unpredictable, with periods of improvement, followed by relapses over extended periods. Managing GPP flares is challenging due to their potential to endanger the patient's life, underscoring the need for treatments that are both fast-acting and highly effective in the case of severe and systematically ill GPP patients. We present a case of a 48-year-old man with an extensive and severe GPP flare (GPP Physician Global Assessment score = 4), experiencing an extensive pustular rash on an erythematous base, intense skin exfoliation, and inflammation as well as systemic symptoms such as fever, hypotension, and general weakness. During the disease course, he developed comorbidities such as depression occurrence and an episode of an acute pulmonary embolism. Initial treatment attempts with acitretin and anakinra were not proved successful. Due to IL-36's significant role in GPP pathophysiology, the patient received treatment involving an IL-36 receptor antagonist (two infusions of 900 mg spesolimab administered one week apart), alongside continued acitretin therapy. This approach led to swift improvement, resolving pustules and skin inflammation and resulting in the patient’s gradual recovery. This case highlights spesolimab's potential as a targeted therapy for severe GPP flares resistant to conventional treatments. However, further research is needed to establish its long-term safety and efficacy in managing GPP and related IL-36-mediated diseases.

## Introduction

Generalized pustular psoriasis (GPP) is an acute, severe form of psoriasis that manifests with painful, yellowish, sterile pustules on an erythematous base over large areas of the body. It is distinct from plaque psoriasis, characterized by unique genetic loci and inflammatory cytokines, with IL-1 and, particularly, IL-36 being predominant [1]. The history of plaque psoriasis in GPP patients varies widely, while a triggering factor can contribute to the progression from plaque psoriasis to GPP [2]. The clinical progression of GPP is typically unstable and is characterized by periods of remission and recurrence over several years. Flares can be triggered by re-exposure to a precipitating factor or may occur for unknown reasons. Patients generally need ongoing treatment to prevent the recurrence of flares. GPP flare can be life-threatening if untreated and may present with serious complications such as high fever, fatigue, malaise, anorexia, dehydration, organ failure, aseptic and hypovolemic shock, and death due to extensive skin barrier disruption, progressive vasodilation, and hypoalbuminemia [3].

Patients with acute GPP often appear systemically ill and typically require hospitalization for adequate supportive care, in terms of their illness severity, vital sign stability, fluid and electrolyte balance, and risk for potential systemic infection. GPP treatment includes systemic therapies, topical therapies, and phototherapy, with systemic treatment preferred initially due to the impracticality of topical therapies for widespread disease. Managing extracutaneous complications, like sepsis or organ dysfunction, is also essential. According to the most recent guidelines, GPP treatments include both non-biologic and biologic medications approved for psoriasis. The sole approved treatment specifically for GPP is spesolimab, an IL-36 receptor antibody. Its intravenous application is authorized for flare treatment in several countries, including the United States, Japan, China, Taiwan, and Canada, while subcutaneous spesolimab has been approved for flare prevention in the United States and China [4]. Regarding non-biologic treatments, acitretin and methotrexate are considered initial treatment approaches for adults with relatively stable GPP [5]. The advantages of those medications are their well-tolerated nature, availability in oral form, and suitability for long-term maintenance. However, their relatively slow onset of action makes them less suitable for patients with more severe diseases, necessitating an alternative approach for such cases. Thus, treating GPP flares is challenging as it can pose significant risks to the patient's life, necessitating a treatment option with rapid onset and high efficacy [3]. Thus, we present a patient with a life-threatening GPP flare who was treated with spesolimab, highlighting the use of an IL-36 receptor antagonist in emergency cases of GPP.

## Case presentation

**Figure 1:**
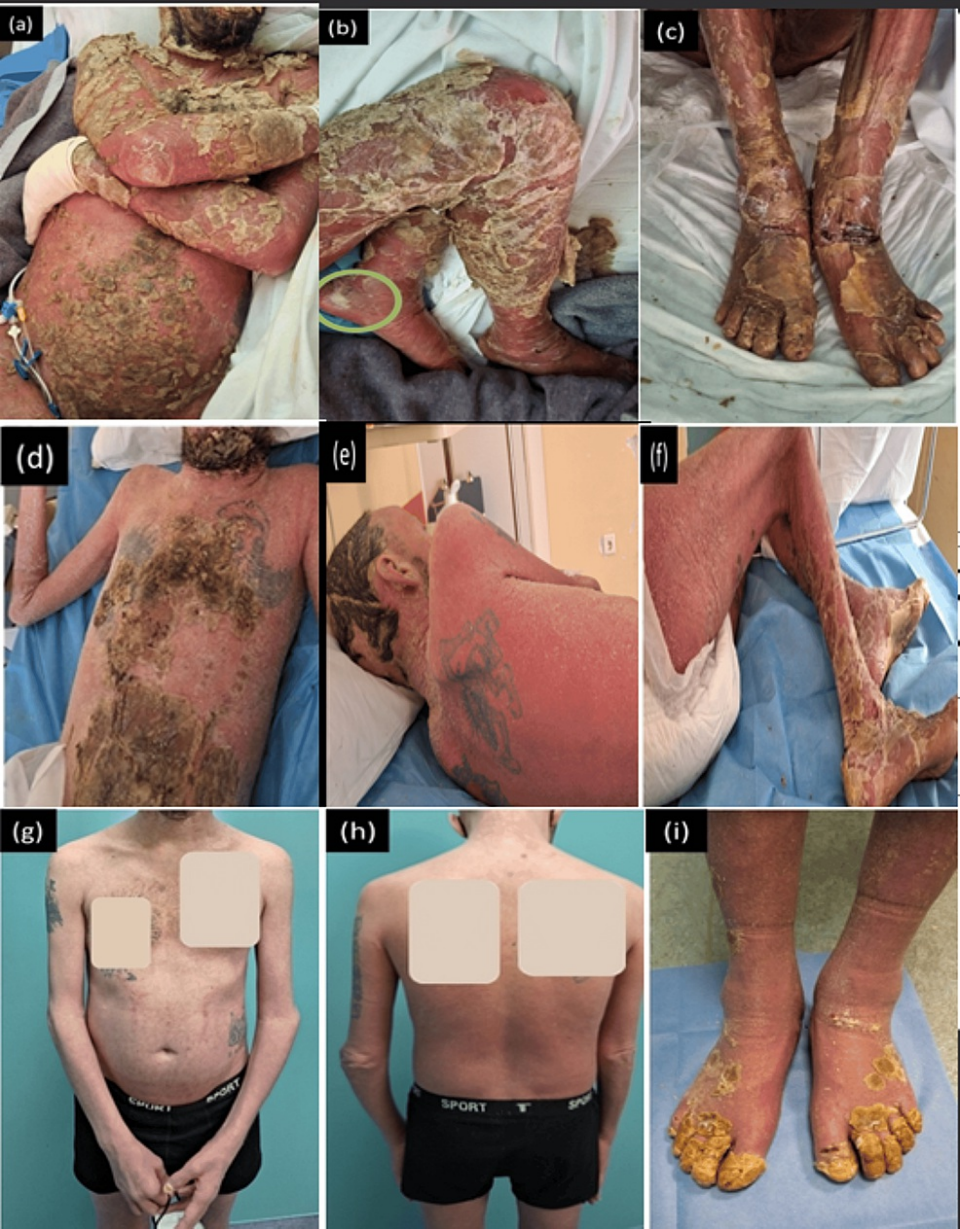
Clinical appearance of the patient. (a-c) The patient at baseline (before spesolimab initiation) presented with pustular rash, severe erythema, and severe desquamation (GPPGA score = 4). (d-f) Seven days after the second infusion of spesolimab 900 mg, the patient had a notable skin improvement, complete clearance of pustules, and mild erythema, while severe desquamation was preserved (GPPGA score = 2). (g-i) Twenty days after the second infusion of spesolimab, the skin of the patient had cleared significantly (GPPGA score = 1). GPPGA: Generalized Pustular Psoriasis Physician Global Assessment.

**Figure 2:**
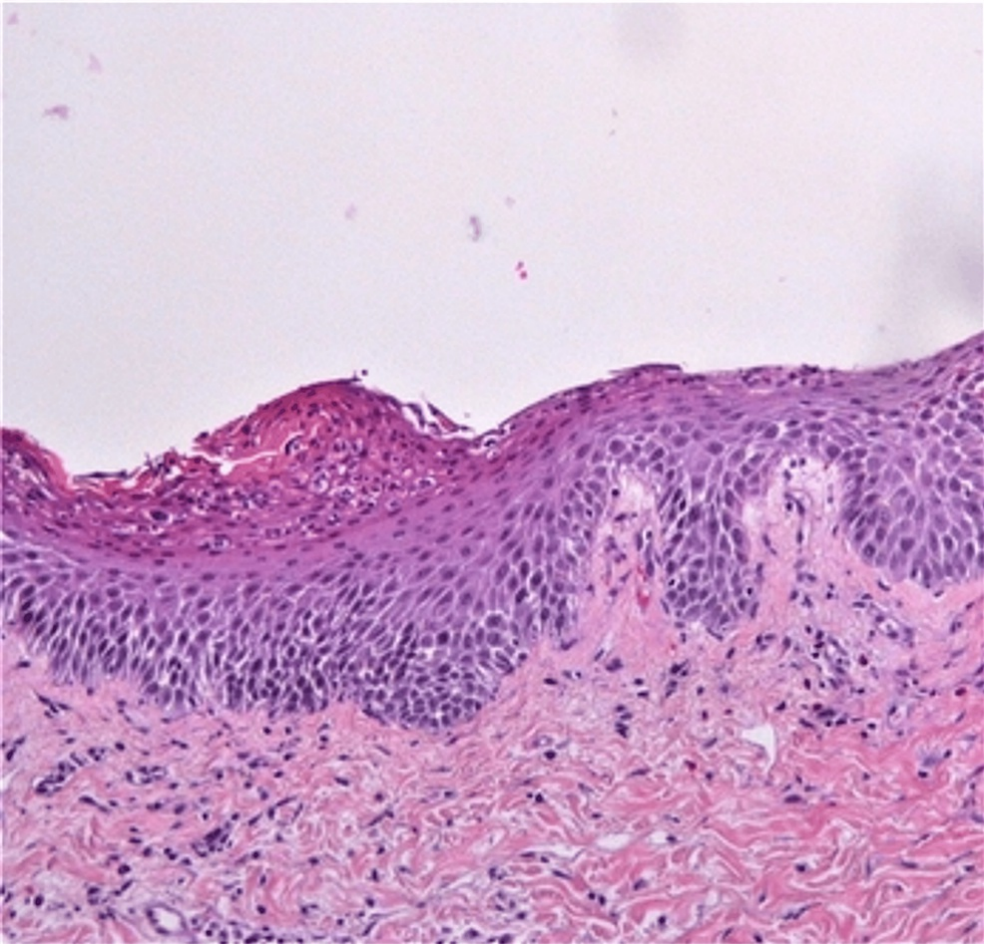
Histopathology of the lesions. Histopathologic picture displaying acanthosis, hyperkeratosis, parakeratosis, loss of granular layer, large Munro abscess, and elongated rete ridges (hematoxylin & eosin staining; original magnification x 100), indicating a histopathology diagnosis of pustular psoriasis.

A 48-year-old male patient with a 20-year history of moderate-to-severe plaque psoriasis vulgaris was admitted to the hospital. He presented with an extensive pustular rash on an erythematous base (Figure 1)(GPP Physician Global Assessment (GPPGA) score = 4), along with a constellation of symptoms including general weakness, fatigue, weight loss, stiffness due to intense skin exfoliation and inflammation, fever (40°C), chills, and hypotension (blood pressure = 100/50 mmHg). He reported a respiratory infection for which he received antibiotics, and 10 days later, he developed a pustular rash on the trunk and extremities. Laboratory findings revealed leukocytosis (13700 10^3^/μL), severe anemia (hematocrit: 26%; hemoglobin: 8.8 g/dL), electrolyte abnormalities, and elevated C-reactive protein (15.9 mg/dL) (Table 1). Further investigation identified *Acinetobacter baumannii* bacteremia. The patient underwent a genetic analysis focusing on the IL36RN gene, which revealed a potentially harmful mutation in exon 5 (heterozygous mutation c.338C>T), mutations lined with GPP. Our diagnosis was GPP superimposed on existing plaque psoriasis vulgaris accompanied by secondary infection, likely resulting from compromised skin integrity due to the severe psoriatic flare. The decision to prioritize GPP over acute generalized exanthematous pustulosis (AGEP) and subcorneal pustulosis in our differential diagnosis was based on the patient's longstanding history of plaque psoriasis vulgaris, the IL-36 mutation’s stronger correlation with GPP, and the absence of immediate pustular rash attribution to drug reactions, which is characteristic of AGEP [3]. A punch biopsy was performed and revealed histopathology consistent with GPP (Figure 2). The patient received systemic intravenous antibiotics based on antibiogram results (cotrimoxazole and ampicillin/sulbactam) for 14 days, along with immunoglobulin G (IV) at 0.4 g/kg/day for five days, antipyretics, and saline hydration. During hospitalization, he developed depression symptoms and was treated with mirtazapine. Left-sided chest pain and elevated D-dimers prompted a CT pulmonary angiogram revealing pulmonary embolism, leading to the initiation of novel oral anticoagulants (NOACs). Acitretin 25 mg/d was administered for 15 days and, due to the central role of IL-1 in the pathogenesis of GPP, anakinra (IL-1 receptor antagonist) IV 100 mg x3 was added without improvement. Topical corticosteroids, moisturizers, and antihistamines managed severe skin dryness, desquamation, and itching. A more disease-focused approach for managing GPP, such as using an IL-36 receptor antagonist, was crucial to address the extensive inflammation in this patient and offer relief for those not responding well to conventional treatments. Thus, we began a 900 mg infusion of spesolimab, resulting in a gradual improvement over one to two days, characterized by resolution of pustules and subsequent erythema and desquamation (GPPGA score = 3). A second infusion of 900 mg spesolimab a week later resulted in significant skin improvement seven days afterward, with complete clearance of pustules, mild erythema, severe desquamation (GPPGA score = 2), and no treatment-related adverse events. Acitretin was discontinued due to severe dryness and scaling, and guselkumab (a monoclonal antibody that targets interleukin-23) was initiated for maintenance therapy as the patient during his plaque psoriasis period has failed to improve with other biologics, such as IL-17 antagonists. The patient remains free of new flare-ups up to now, i.e., a period of five months.

**Table 1:** Patient's laboratory abnormalities. Table presenting the laboratory findings of our patient experiencing a severe GPP flare. The exams indicate leukocytosis, severe anemia, and high CRP. GPP: generalized pustular psoriasis; HCT: hematocrit; Hb: hemoglobin.

Patient laboratory exams		Normal range
Leukocytosis	WBC: 13,700 10^3^/μL	WBC: 4,500-11,000 10^3^/μL
Severe anemia	HCT: 26%; Hb: 8.8 g/dL	HCT: 41%-53%; Hb: 13.5-17.5 g/dL
Elevated markers of inflammation	C-reactive protein: 15.9 mg/dL	C-reactive protein: <0.3 mg/dL

## Discussion

Spesolimab is a humanized monoclonal IgG1 antibody that specifically binds to the IL-36 receptor with high affinity, preventing ligands (IL-36α, β, and γ) from activating IL-36 receptor and blocking downstream activation of proinflammatory pathways [6]. Therefore, it has a more targeted approach as the IL-L36-chemokine-neutrophil axis appears to be central to the pathogenesis of GPP. Biological treatments such as guselkumab [7], anakinra [8], and infliximab (anti-tumor necrosis factor) [3], as well as non-biological options such as ciclosporin [5], have proven effective for controlling GPP within a week, preventing further complications from severe flares. As observed in our case, spesolimab shows a quick response, with its effects usually becoming noticeable within one to three days, as evidenced by the clearance of pustules.

A clinical trial has shown spesolimab to be safe and effective for GPP patients even without IL-36 mutations [9]. This study demonstrated that a high-dose subcutaneous regimen of spesolimab (600 mg loading dose, followed by 300 mg every four weeks) is superior to a placebo in preventing GPP flares over 48 weeks [9]. In a randomized clinical trial including adults with moderate to severe GPP flares, a single 900 mg subcutaneous dose of spesolimab vs. placebo was compared on their response. By day eight, 54% of patients in the spesolimab group achieved a GPPGA score of 0 compared to only 6% in the placebo group who achieved clearance. Side effects reported were infections (at week 12) and DRESS (drug reaction with eosinophilia and systemic symptoms) syndrome occurrence, with less frequent drug interactions, including drug-induced hepatic injury [10].

Spesolimab has also been used as a treatment approach for a patient with co-existing GPP and bullous pemphigoid, highlighting the potential of IL-36 inhibition not just for managing pustular psoriasis, but also for offering a safer treatment option for patients with concurrent autoimmune skin disorders [11]. Apart from GPP, spesolimab has been used as a treatment for a patient with pyoderma gangrenosum with the disease resolving within 72 hours of infusion, suggesting a direct effect of IL-36 antagonist as this cytokine plays a key role in the inflammatory cycle of this disease [12]. Therefore, spesolimab has been proven to be a promising treatment for neutrophilic diseases, though further studies are needed to assess its long-term safety and establish it as a new treatment option for such patients.

## Conclusions

Spesolimab seems to be an important therapeutic tool for managing rapidly progressing severe GPP flare as it has a highly selective mode of action, while it seems to be effective when GPP flare poses a life-threatening risk and does not respond to other medications. Spesolimab exhibits potential as a treatment for diseases where IL-36 plays a significant role in their pathophysiology. However, additional studies are necessary to evaluate its long-term safety and establish it as a viable treatment option for such patients.

## References

[1] Johnston A, Xing X, Wolterink L (2017). IL-1 and IL-36 are dominant cytokines in generalized pustular psoriasis. J Allergy Clin Immunol.

[2] Karampinis E, Papadopoulou MM, Chaidaki K (2024). Plaque psoriasis exacerbation and COVID-19 vaccination: assessing the characteristics of the flare and the exposome parameters. Vaccines (Basel).

[3] Rivera-Díaz R, Daudén E, Carrascosa JM, Cueva P, Puig L (2023). Generalized pustular psoriasis: a review on clinical characteristics, diagnosis, and treatment. Dermatol Ther (Heidelb).

[4] Choon SE, van de Kerkhof P, Gudjonsson JE (2024). International consensus definition and diagnostic criteria for generalized pustular psoriasis from the International Psoriasis Council. [PREPRINT]. JAMA Dermatol.

[5] Zhou LL, Georgakopoulos JR, Ighani A, Yeung J (2018). Systemic monotherapy treatments for generalized pustular psoriasis: a systematic review. J Cutan Med Surg.

[6] Burden AD (2023). Spesolimab, an interleukin-36 receptor monoclonal antibody, for the treatment of generalized pustular psoriasis. Expert Rev Clin Immunol.

[7] Sano S, Kubo H, Morishima H, Goto R, Zheng R, Nakagawa H (2018). Guselkumab, a human interleukin-23 monoclonal antibody in Japanese patients with generalized pustular psoriasis and erythrodermic psoriasis: efficacy and safety analyses of a 52-week, phase 3, multicenter, open-label study. J Dermatol.

[8] Karampinis E, Gravani A, Gidarokosta P, Bogdanos DP, Roussaki-Schulze AV, Zafiriou E (2023). Pustular eruption following COVID-19 vaccination: a narrative case-based review. Vaccines (Basel).

[9] Morita A, Strober B, Burden AD (2023). Efficacy and safety of subcutaneous spesolimab for the prevention of generalised pustular psoriasis flares (Effisayil 2): an international, multicentre, randomised, placebo-controlled trial. Lancet.

[10] Bachelez H, Choon SE, Marrakchi S (2021). Trial of spesolimab for generalized pustular psoriasis. N Engl J Med.

[11] Teshima R, Saito-Sasaki N, Hitaka T, Sawada Y (2024). Spesolimab in the management of generalized pustular psoriasis with concurrent bullous pemphigoid and psoriasis. Cureus.

[12] Guénin SH, Khattri S, Lebwohl MG (2023). Spesolimab use in treatment of pyoderma gangrenosum. JAAD Case Rep.

